# Developing emotional intelligence in a static and interactive music learning environment

**DOI:** 10.3389/fpsyg.2024.1279530

**Published:** 2024-02-05

**Authors:** Jitao Liang

**Affiliations:** Conservatory of Music, Huanggang Normal University, Huanggang, Hubei, China

**Keywords:** emotional intelligence course, identifying emotions, long-term perspective, managing emotions, Mayer-Salovey-Caruso model

## Abstract

The concept of a static electronic learning environment implies the utilization of tools that do not necessitate any active participation on the part of the learner aside from familiarizing oneself with the provided materials. In contrast, an interactive electronic learning environment necessitates active engagement and interaction from the learner. The research purpose is to compare the impact of interactive and static learning environments on students’ emotional intelligence. Music education students (*n* = 216) took a theoretical course on emotional intelligence during one academic semester at the university. The students were randomly divided into two equal groups: Group A (*n* = 108) attended the course in a lecture format, using videos and additional reading materials. Group B participated in online forums, completed interactive exercises, and discussed individual personal diaries kept at home with the teacher. At the end of the semester, both groups completed the Mayer-Salovey-Caruso emotional intelligence test. The results suggest that the total score for emotional intelligence was higher in Group B. After another 6 months of training, students passed the same test again to assess the effectiveness of the long-term intervention strategy. The post-training results suggest that Group B has an advantage in training over Group A. At the same time, in the Managing Emotions subcategory, Group B was behind Group A, but after 6 months of training, Group B significantly improved the results (value of *p* = 0.01). The research summarizes the practical significance of the theoretical course on the development of emotional intelligence among music education students.

## 1 Introduction

Learning music is a complex process that requires a well-developed theoretical background and regular practice, constant interaction, good feedback, and intrinsic motivation (McGinnis, 2018). The rapid development of technology has profoundly influenced musicians and teachers of music education. The main advantages of the proposed approach involve better academic achievements, engagement, and general interest in music among higher education students (Lv and Luo, 2021). Not all strategies aimed at enhancing student learning are equally effective. Therefore, Hattie (2023) advocates for the identification and implementation of strategies that yield significant effects.

The research focuses on a comparison between the traditional academic approach and the use of modern online technologies (Stevens et al., 2021; Wang, 2022).

Modern technology helps students to compare their academic achievements with the results of individuals and interact with each other and the teacher. This approach should cause deeper reflection and analysis (Hanrahan et al., 2019). Modern music education researchers use the terms Digital technologies, Interactive learning environment, Innovation, Digital learning tools, and Mobile learning in their works.

Liu et al. (2023), for example, define mobile learning as a pedagogical model in which learning is facilitated by mobile technologies. In addition, the researchers specify that students who have access to mobile devices (phones or tablets) improve significantly learning outcomes (Liu et al., 2023). At the same time, Liu et al. (2023) provide information about an interactive whiteboard that helps students learn the basics of music. The scholars focus on what mobile technologies mean for education and whether mobile technologies have antipodes in the electronic educational environment.

The present research, as well as the work of Zhang (2022), proposes to differentiate the e-learning environment between interactive (mobile) and static environments. The proposed differentiation is based on the analogy with interactive and static graphics (Weissgerber et al., 2016; Ismaeel and Al Mulhim, 2021) as well as interactive and static content (Balážiová, 2019; Masson et al., 2020). In this research, a static learning environment means e-learning tools that do not require any actions from the learner but to review the proposed materials. At the same time, when the learning environment requires interaction, manipulation, and feedback, it will become an interactive e-learning environment.

Modern research generally agrees that cognitive skills are not the only factor that influences academic success in education and professional activity. Emotional competencies also play a key role in knowledge acquisition and should be systematically analyzed by scientists (Nelis et al., 2009).

The concept of emotional intelligence (EI) means human skills required to interact with others and control personal emotions. The existing tests for General Intelligence are less effective for EI. Thus, researchers perceive EI as an integral part of life success and overall happiness (Nelis et al., 2009). However, the modern approaches to the assessment of emotional competencies differ significantly.

EI means the ability to understand the intentions, desires, and motivations of an individual and peers as well as to recognize and express emotions in the right way (Megías-Robles et al., 2020). Contemporary students routinely leverage digital technologies, including e-learning, as a means of acquiring knowledge. In certain instances, digital education entirely supplants traditional instructional methods. The trend of integrating digital technologies into everyday educational practices intensifies each year (Schmidt and Tang, 2020; Facer and Selwyn, 2021). In this context, a crucial objective emerges: to compare the effectiveness of various electronic learning modalities (interactive and static) on distinct components of student success. Furthermore, empirical evidence in the literature supports the hypothesis that a significant correlation exists between EI and students’ performance (Pool and Qualter, 2012; Allen et al., 2014; McGinnis, 2018).

Given the significance of EI and the limited exploration of EI within the literature in the context of electronic learning environments, this study aims to address this gap. The ensuing analysis involves the evaluation of fundamental EI models. In the experimental phase, the EI model, derived from a comprehensive literature review, will be assessed within both static and interactive learning environments.

### 1.1 Mayer-Salovey-Caruso ability model

The modern literature discusses three types of EI models: ability model, mixed model, and trait model (Kanesan and Fauzan, 2019). The core model of emotional intelligence used to assess emotional skills is the Mayer-Salovey-Caruso model. The model focuses on four aspects: perception of emotions, use of emotions, understanding of emotions, and management of emotions (Megías-Robles et al., 2020). These four aspects represent a set of emotions, including mental abilities, and that is measured from the beginner to more advanced levels (Kanesan and Fauzan, 2019). The Mayer-Salovey-Caruso model excludes personal and other competencies and describes the model of emotional intelligence only.

The Mayer-Salovey-Caruso ability model is popular in many modern pieces of research (Nelis et al., 2009; Pool and Qualter, 2012; Corcoran and Tormey, 2013). The uniqueness of this approach is not only in the framework of the four categories of EI perceived as an ability, but also in the Mayer-Salovey-Caruso emotional intelligence test (MSCEIT), which requires participants to solve problems related to emotions, and not just answer questions as similar EI tests ask respondents to do. In addition to MSCEIT, the ability model uses other assessments to ensure high-quality results. In the research by Nelis et al. (2009), EI is assessed using six different questionnaires. In addition to the EI questionnaire, an emotion profile questionnaire, an emotion management ability test, the Toronto Alexithymia Scale, and a situational emotion understanding test are used.

### 1.2 Modern education and emotional intelligence

The development of EI has become a key issue in modern education. High EI has a significant impact on higher academic and later professional achievements (Jiménez-Picón et al., 2021) and helps to cope with negative emotions that lead to failures (Weissberg, 2000). Moreover, EI reduces the potential risk of bad habits development, mental illness, and delinquency problems (Kam et al., 2003). EI is associated with social competence, self-esteem (MacCann et al., 2011), susceptibility to bullying, and intimidating behavior toward others (Lomas et al., 2012).

The relationship between EI and academic performance is of concern to many researchers. MacCann et al. (2011) report that EI predicts the academic performance of K–12 students, the grade point average in universities, and student attrition from the university. A recent meta-analysis on this topic (Akpur, 2020; MacCann et al., 2020) provides a significant positive impact of EI on academic performance. Sekreter (2019) notes that EI is more important for life success than IQ. MacCann et al. (2020) suppose that a low level of EI indicates a student’s weak adaptive ability, inability to cope with stress, susceptibility to social pressure, and increased anxiety. Several scientists have found only an insignificant relationship between academic achievement and EI (Ranjbar et al., 2017). They explain it by the lack of attention of educational professionals to the development of EI (Olatoye et al., 2010; Ranjbar et al., 2017).

Researchers in music education often correlate personal skills associated with EI with success in teaching, stress management, and the ability to communicate positively (Miksza et al., 2010; MacLeod and Walter, 2011). It is reported that social interaction skills and socio-emotional intelligence are crucial for participation in musical programs (Kaschub, 2002).

McGinnis (2018) investigated the correlation between EI and academic performance following the implementation of strategies from EI in bachelor of music education programs. McGinnis’s (2018) sample comprised 10 students enrolled in a semester-long course on the fundamentals of music teaching. When students were asked to predict their outcomes, their forecasts were notably inflated. However, post-training results indicated improvements in EI attributed to the completed course. McGinnis (2018) supposes that further research is needed to test the tools used to measure EI and determine the effectiveness of using pedagogical interventions to improve EI scores. Humphrey et al. (2007) admit that modern education should assess the long-term consequences of introducing EI development courses for students.

Corcoran and Tormey (2013) focused on examining the relationship between EI and the performance of student teachers. With a sample of 352 student teachers, they sought evidence indicating that higher EI corresponds to effectiveness in teaching. However, according to the findings of Corcoran and Tormey (2013), those student teachers who scored higher on emotional self-awareness and awareness of others had lower performance ratings. The correlation between EI and performance was identified as weak to moderate. Corcoran and Tormey (2013) attribute their results to the absence of outcome-based teaching assessments that would encompass attention to the relational and emotional aspects of the teaching role. The teacher-student relationship, along with the personal qualities of the teacher, are acknowledged as crucial factors in education and can contribute to the overall learning experience (Hattie, 2023).

The present research aims to fill these knowledge gaps. The main research goal is to compare the impact of interactive and static learning environments on EI.

The primary research hypotheses are as follows:

*H1*: A dynamic learning environment is more effective in fostering Emotional Intelligence (EI) development than a static one.

*H2*: The development of Emotional Intelligence (EI) through the utilization of a dynamic environment will yield a longer-lasting impact compared to the utilization of a static environment.

## 2 Research methodology

### 2.1 Participants

At Kharkiv National University of Arts named after I. P. Kotlyarevsky., Ukraine, the second-year students undertook an optional mixed course in emotional intelligence. The training lasted for 6 months (1st academic semester of 2021). The sample involved 216 students who voluntarily agreed to participate in the research. Students of instrumental (*n* = 125) and choral (*n* = 91) music education were randomly divided into equal groups to learn in the static (Group A, *n* = 108) and interactive (Group B, *n* = 108) learning environments. All participants were adults; the average age was 20.4 years (61% females and 49% males).

### 2.2 Methods

Each group had one lesson every 2 weeks at the university. Each lesson lasted for 1.5 h. The interval of 2 weeks ensured an opportunity for participants to apply the knowledge and skills they had acquired during the emotional intelligence course to their academic activities and daily life. The lesson consisted of a lecture. Each lecture involved a presentation on an interactive whiteboard, and students had the opportunity to ask questions. Educators provided each group with the links to home-reading and video-watching materials to review after the academic session. In addition, Group B discussed personal diaries, e-discussions, and interactive exercises available on the network. Only Group B kept a personal diary in which the participants noted their emotional experiences. A special group was created in one of the popular social networks for students to share and discuss the content that aroused certain emotions (news, posts, photos, and videos).

At the end of the semester, the emotional experience of the participants in both groups was assessed using the EI test (MSCEIT-1). The long-term impact of EI training, according to (Nelis et al., 2009), was re-assessed after another 6 months of training and involved those students who took the emotional intelligence (MSCEIT-2) course before.

### 2.3 Instruments

The Ukraine version of MSCEIT was selected as the research instrument to ensure high results validity (Cronbach mean alpha = 0.85) (Mao et al., 2016). According to Mayer (2002), MSCEIT measures an individual’s total EI score and covers two domains: Experiential emotional intelligence (EEIQ) and Strategic emotional intelligence (SEIQ).

**Figure 1:**
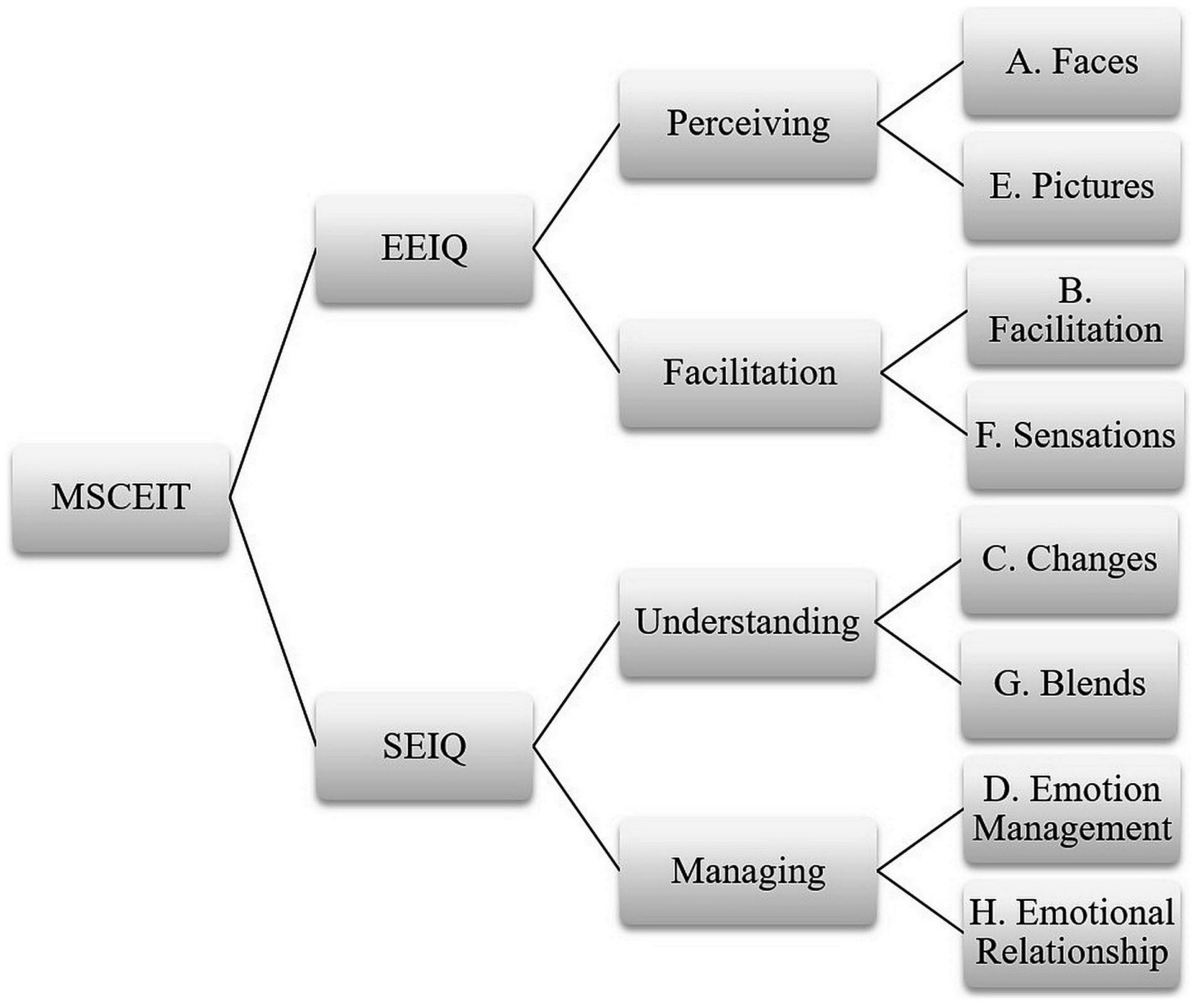
MSCEIT scheme.

Each category contains two subcategories. EEIQ involves categories such as: (1) Perceiving and Identifying Emotions: the ability to identify emotions, personal emotions, and emotions of others; (2) Facilitation of Thought: the ability to generate and discuss emotions and compare them with each other as well as sensations and thoughts of other individuals. SEIQ includes domains as follows: (1) Understanding Emotions: the ability to understand complex emotions, trace emotional chains, and discuss emotions; (2) Managing Emotions: the ability to regulate personal emotions and the emotions of others, empathize, relieve anxiety for oneself and others. The test scheme is available in Figure 1.

There are separate tasks for each subcategory. The tasks are available in Table 1. The reliability of the scale in this study was deemed satisfactory, with a Cronbach’s alpha 0.79.

**Table 1:** Examples of MSCEIT tasks (compiled by the author based on Mayer, 2002).

EEIQ (1)	Perceiving and identifying emotions	Describe the facial emotion in the photo	Happiness	1 2 3 4 5
			Fear	1 2 3 4 5
			Sadness	1 2 3 4 5
EEIQ (2)	Facilitation of Thought	What mood(s) might be helpful to feel when meeting future mother-in-law for the first time? 1 – Not Useful, 5 – Useful.	Tension	1 2 3 4 5
			Surprise	1 2 3 4 5
			Joy	1 2 3 4 5
SEIQ (1)	Understanding Emotions	Tom was worried that he had much work to do. When the manager gave him an additional project, he felt (Choose the best option)	(a) Overwhelmed	
			(b) Depressed	
			(c) Ashamed	
			(d) Self Conscious	
			(e) Jittery	
SEIQ (2)	Managing Emotions	Debbie, calm and contented, has just returned from vacation. Rate the effectiveness of each of her actions (1 – Very unproductive, 5 – Very productive)	She started making a to-do list	1 2 3 4 5
			She started planning her next vacation	1 2 3 4 5
			She ignored the feeling since it would not last	1 2 3 4 5

### 2.4 Statistical data analysis

The normality of distribution was assessed based on skewness and kurtosis values. The skewness and kurtosis values did not exceed 1 (Skewness 0.69, kurtosis 0.56). Consequently, the data exhibited a normal distribution, aligning with the requirements for parametric tests (Pallant, 2020). The homogeneity of variance was examined using Levene’s test. The groups’ results were compared using Student’s *t*-test. The temporal changes in the results of each group were assessed. IBM SPSS Statistics 23 was used for this purpose.

## 3 Results

The *t*-test results of the two groups after the optional six-month course on emotional intelligence are presented in Table 2. Group B provides a higher level of EI: the average between the EI scores for Group A: Maver = 0.325, and for Group B: Maver = 0.454. A statistically significant advantage of Group B is observed in both subcategories of Perceiving and Identifying Emotions, Facilitation of Thought, related to Experiential emotional intelligence. For the Understanding subcategory of Strategic emotional intelligence, no statistically significant differences between the two groups were identified. Differences in Emotional Relationships were found in Group A, which can be explained by the lecture course, video viewing, and reading additional materials. In addition, a small but statistically insignificant advantage of Group A in Emotion Management was identified by the test.

**Table 2:** Descriptive *t*-test statistics between Group A and Group B after the course (MSCEIT-1).

		Group A, *n* = 108		Group B, *n* = 108				
Subcategory MSCEIT	Task level	*M*	SD	*M*	SD	*t*	Value of *p*	*d*
Perceiving	A. Faces on photographs	0.292	0.22	0.372	0.36	2.28	0.011	0.388
	E. Pictures	0.311	0.41	0.344	0.45	3.04	0.026	0.077
The average for subcategory		0.302	0.315	0.358	0.41			
Facilitation	B. Facilitation	0.354	0.48	0.402	0.51	2.56	0.022	0.275
	F. Sensations	0.313	0.26	0.364	0.36	3.31	0.015	0.324
The average for subcategory		0,334	0.370	0.383	0.48			
Understanding	C. Changes	0.306	0.34	0.291	0.49	−1.07	2.105	–0.036
	G. Blends	0.288	0.41	0.302	0.38	1.12	0.196	0.035
The average for subcategory		0.297	0.375	0.297	0.43			
Managing	D. Emotion Management	0.313	0.52	0.291	0.45	0.96	0.274	−0.045
	H. Emotional Relationship	0.341	0.39	0.286	0.54	2.19	0.001	0.387
The average for subcategory		0,327	0.455	0.289	0.50			
Average score		0,314	0.372	0.335	0.441			

The tasks of the Perceiving subcategory are to identify emotions in the human faces depicted in the photographs and pictures. Individuals who receive low scores in perceiving emotion have difficulty identifying the emotions of others.

The inability to understand the emotions of other individuals leads to an inability to manage emotions in different situations (Mayer, 2002). The performance of musicians is connected with communication, including interaction with the audience. The research results suggest that the average for the Perceiving subcategory for Group A was 0.302, while for Group B it was 0.358 (Table 2) on the emotional intelligence course. Moreover, the research found that Group B better perceives its own emotions and the emotions of the audience and is more skillful in exchanging energy with the audience (Perceiving subcategory). Hsia and Sung (2020) suggest that the more convincing the musician’s relations with the audience, the better his high-performance skills.

In the sensations (empathy) tasks of MSCEIT, emotions were compared with concepts of light, color, and temperature. At the end of the emotional intelligence course, Group B of the interactive learning environment provided an average of MGroupB = 0.364 in this subcategory. This result differs from the static learning environment (Group A), which scored on average for the subcategory MGroupA = 0.334. The lower scores in Group A indicate the difficulty experienced by participants in generating emotions and comparing and contrasting them with sensory modalities. Group B provided better results on this test.

The tasks provided in the Understanding Emotions subcategory consisted of building chains of emotions and assessing the ability to transfer simple emotions into complex ones. After the course, groups A and B revealed no significant differences in this subcategory. A high score indicates strong emotional insight. According to Mayer (2002), a low score specifies the inability of an individual to recognize the emotions of others if he reacts differently to the event. The results (Table 2) suggested that emotional insight in Group A was slightly higher in the Blends task level, while emotional insight of Group B was slightly higher in the Changes task level.

The Managing subcategory evaluated the impact of actions on the result with the involvement of other individuals. Respondents who misinterpreted the situation and blamed others, as well as felt hurt and offended, scored lower. Both groups had approximately equal results regarding Emotion Management, but the results were not high. The analysis of Emotional Relationships (Table 2) suggested that musicians from the static learning environment outperformed their colleagues from the interactive learning environment and proved strong social bonding skills. Thus, the research found that musicians had to learn to think about events from different points of view, despite emotional distress, regulate their emotions, and make balanced emotional decisions.

MSCEIT was conducted for the second time after 6 months of the emotional intelligence course. Table 3 is a comparison of the results for both groups.

**Table 3:** Descriptive statistics of the *t*-test between Group A and Group B 6 months after the course emotional intelligence (MSCEIT-2).

		Group A, *n* = 108			Group B, *n* = 108			Value of *p* (Group A – Group B)	*d* (Group A – Group B)
Subcategory MSCEIT	Task level	*M*	SD	Value of *p* (Test1-Test2)	*M*	SD	Value of *p* (Test1-Test2)		
Perceiving	A. Faces	0.305	0.45	2.960	0.366	0.41	1.110	1.263	0.042
	E. Pictures	0.319	0.52	1.150	0.351	0.55	2.016	−1.46	0.06
The average for subcategory		0.312	0.49	2.055	0.359	0.48	0.064		
Facilitation	B. Facilitation	0.336	0.59	0.512	0.421	0.37		0.001	0.296
	F. Sensations	0.315	0.41	−1.347	0.332	0.28	0.01	0.001	0.288
The average for subcategory		0.326	0.50	−0.930	0.377	0.31	0.089		
Understanding	C. Changes	0.296	0.33	2.033	0.298	0.48	1.197	1.456	0.005
	G. Blends	0.254	0.26	0.03	0.322	0.39	0.021	0.006	0.266
The average for subcategory		0.275	0.30	2.033	0.310	0.44	0.505		
Managing	D. Emotion Management	0.273	0.37	0.02	0.326	0.42	0.020	0.012	0.254
	H. Emotional Relationship	0.288	0.25	0.004	0.319	0.51	0.003	0.01	0.248
The average for subcategory		0.281	0.31		0.323	0.47			
Average score		0.298	0.40		0.342	0.43			

Table 3 provides the information that both groups have statistically significant differences in the Managing subcategory. In this subcategory, groups from the static learning environment received decreased scores in MSCEIT-2 compared to MSCEIT-1 (Maver1 = 0.327, Maver2 = 0.298), while the interactive learning environment group, on the contrary, improved the results (Maver1 = 0.289, Maver2 = 0.323). This may indicate a more delayed effect of the implemented course in the interactive learning environment on the development of Emotion Management and Emotional Relationships. Immediately after the course, Group B received lower results compared to Group A, but they improved later. The researchers admitted that the acquired knowledge transferred into skills that were gradually developed during the next months. Group B became more productive in making emotional decisions.

After the course, in the subcategories Perceiving and Facilitation the respondents scored higher in Group B. This advantage and skills remained stable for half a year. In the Understanding subcategory, Group B had higher results in half a year, while Group A, on the contrary, had lower results. Six months after the course, the average score for EI of Group A was 0.298, and Group B was 0.342.

## 4 Discussion

The research results indicate that a dynamic learning environment is more effective for the development of Emotional Intelligence (EI) than a static one (H1). After the course and in a 6-month interval, students in the interactive learning environment demonstrated higher EI skills (H2). Test 1 suggested that the advantage of students in the static environment group on Emotional Relationships was short-term and decreased in 6 months. Thus, the research concludes the following: (1) IE can be developed; (2) after the intervention, the acquired competencies can be developed to a higher level by practicing them regularly; and (3) a dynamic learning environment is more effective than a static one for the development of Emotional Intelligence (EI).

Conclusion (1) aligns well with previous studies by Nelis et al. (2009), McGinnis (2018), and Năstasă et al. (2023), which have documented the effectiveness of interventions aimed at enhancing Emotional Intelligence (EI). McGinnis (2018) offered the course known as the Intelligence 2.0 Method to music education students. Method 2.0 by McGinnis (2018) involved a printed book and access to two online resources on emotional intelligence. Each participant in McGinnis’s (2018) study selected an action plan, an EI skill for improvement, three strategies recommended for the development of that specific skill, and a mentor who would oversee the process. McGinnis (2018) found that students overestimated their level of EI before commencing their training. Additionally, male participants scored higher than female participants, and participants in choral education achieved higher scores compared to those in instrumental education The test revealed the progress in the development of EI among all participants. The findings of McGinnis (2018) exceeded the primary research expectations. Năstasă et al. (2023) delineated the implementation experience of emotional intelligence development program comprising eight exercises among a sample of teenagers and adolescents aged 16 and 19 years. The EI program described by Năstasă et al. (2023), was grounded in techniques inspired by expressive creative psychotherapy. Following the intervention, Năstasă et al. (2023) recorded significant growth in three out of the four EI domains: Perceiving, Facilitation, and Managing. In our study, participants in the dynamic learning environment exhibited higher scores in these three categories after completing the course, and after 6 months, they additionally demonstrated an advantage in Understanding. In both studies, McGinnis (2018) and Năstasă et al. (2023) implemented interventions that were more aligned with a dynamic rather than a static learning environment as characterized in this research. The fact that Emotional Intelligence (EI) can be developed holds significant implications for music education, given that musicians are individuals immersed in the realm of social interaction. Typically, musicians engage in collaborative rehearsals where they synchronize and discuss forthcoming performances. The ability to regulate one’s own emotions and be aware of how another team member perceives and responds to criticism becomes a crucial skill for the teamwork of musicians (Kaschub, 2002).

Conclusion (2) corroborates the study by Nelis et al. (2009), where positive changes in the development of EI are preserved 6 months later. Moreover, the scholars specify that the training results immediately after the course and 6 months later may increase. The results obtained by Nelis et al. (2009) documented a change in the identification and management of individual emotions and the emotions of others after IE training. The scholars reported no change in understanding and expression of emotions. The present research revealed no progress in the Understanding emotions subcategory in the 6-month interval in Group A. The progress was observed in Group B. The intervention proposed by Nelis et al. (2009) proposed the same definition of an interactive learning environment as the present research. In the Facilitation subcategory, the interactive learning environment group had a statistically significant regression after 6 months of training.

As for Conclusion (3), unfortunately, no specific studies were found regarding the comparison of the effectiveness of dynamic and static learning environments in higher education for the development of Emotional Intelligence (EI). Iaosanurak et al. (2016) discuss the effective implementation of technology for school-age children to educate them about emotional learning. For Iaosanurak et al. (2016), technology is a catalyst and support tool to involve learners in collaborative activities, dialogue, problem-solving, decision-making, and reflection practices, that is, a dynamic environment, as confirmed by the findings of this study. Khasawneh (2018) addresses the impact of EI on the introduction of technology for employees across companies. Khasawneh (2018) reports that participants with a high degree of technology acceptance have a stronger relationship with EI, which is also indirectly consistent with the present findings on the benefits of an interactive learning environment for the development of EI. New teaching methods that emphasize EI in the electronic learning environment may prompt musician students to become aware of their emotions while performing, creating, or listening to musical compositions. Consequently, this awareness could enable musician students to gain a deeper understanding of the relationship between emotions and music, which, fundamentally, constitutes a primary objective of music education (Kaschub, 2002).

At the same time, Sun and Nembhard (2023) examined the interplay between learning representation, arousal, engagement, and learning outcomes using two instructional representations (static and dynamic). In the study by Sun and Nembhard (2023), participants were required to learn letters representing semaphore flags using two representations: video (dynamic representation) and graphic depiction (static representation), followed by an assessment following the instruction. Sun and Nembhard (2023) observed that the static representation was associated with a more stable level of arousal and a lower peak interaction level, both of which were correlated with better performance. In our study, the dynamic learning environment proved to be more effective for Emotional Intelligence (EI) development; while the results differ from those of Sun and Nembhard (2023), it is essential to acknowledge that the experimental tasks in their study were fundamentally different. This underscores the necessity for further comparative research on the impact of dynamic and static environments, particularly on the emotional intelligence of students. Practical value of the course for students.

The results of MSCEIT-1 and MSCEIT-2 suggest that the interactive electronic environment is effective for the development of EI among music education students. What were the advantages for students developing their EI for professional activities? Firstly, the students will understand and perceive the audience better, be able to respond to their moods and desires, and therefore perform better at work and in life.

Secondly, students learned to manage and accept the emotions of others, which may differ from their emotions. A musician or music teacher has to play a piece of music with an emotional background that differs from his perception of the world. Thirdly, students practiced the skills and mastered how to manage their emotions. This skill is significant for musicians who may make a mistake during the performance and be strong emotionally to continue the music program. Fourthly, a well-developed, stable EI will allow musicians and music teachers to be the aesthetic remedy for the viewer, reducing anxiety and giving relaxation and joy. Music education students who work on their EI can achieve positive results for personal growth, professional development, social well-being, mental health, and productivity (Nelis et al., 2009).

### 4.1 Limitations and further research

The research has several limitations. Both groups attended extracurricular training courses, which impacted the final results. The research covered only one age group. All participants were students of one educational institution only. The personal characteristics of participants were not compared using the selected model of EI (Mayer et al., 2007). Thus, the scientists admit the possibility that personal characteristics may influence EI. Future research is needed to analyze the relationship between EI and personal characteristics (for example, extraversion and introversion). A need exists to expand the geography of MSCEIT research, as its developers note that cultural differences can cause differences in EI (Mayer et al., 2007).

The research highlights that EI is an important and necessary competency that should be carefully investigated by scholars. Individuals with high levels of EI have hypersensitivity, which leads to increased susceptibility and psychological well-being (Nelis et al., 2009). The research paid no attention to the testing of the negative impact of EI. Further research is required to investigate this issue.

## 5 Conclusion

The main research goal was to compare the impact of interactive and static learning environments on EI. In the static environment, students received theoretical material in the lecture format presented by the teacher and read and reviewed additional materials. One part of the students received only this knowledge (Group A). Another part of the students (Group B) entered an interactive electronic environment and participated in online events, discussing personal diaries with the teacher, which they kept at home. The results confirm the effectiveness of the interactive environment for the development of EI among students. Thus, the research hypothesis H1 has been confirmed. Six months post-intervention, students in the dynamic learning environment (Group B) exhibited an overall higher level of Emotional Intelligence compared to students in the static learning environment (Group A). This indicates the validation of research hypothesis H2.

Integration of additional courses into the educational process, similar to the experience described above, is an important decision, which helps students achieve mastery of emotional management and maintain positive relations with others. Moreover, the proposed approach will help students to manage and identify their emotions, apply skills of emotion management, control emotions, and build emotional chains and cause-and-effect relationships. Through EI assessment tools (particularly the MSCEIT), students have been presented with the opportunity to assess their emotional abilities and reveal their emotional potential. It will have a positive impact on future careers and social relations. This research provides teachers and administrators of music education with the concept and components of EI and describes the introduction of the course for the development of EI in music education. Moreover, the research provides information on the effectiveness of the proposed approach from short-term and long-term perspectives for students.

### 5.1 Ethical issues

The respondents participated in the research voluntarily and without remuneration. All participants were informed about the research objectives and procedure. No personal information was collected or stored. The research procedure was approved by the ethics committee of Huanggang Normal University (protocol TR 45967818).

## Data availability statement

The raw data supporting the conclusions of this article will be made available by the authors, without undue reservation.

## Ethics statement

The studies involving humans were approved by ethics committee of Huanggang Normal University. The studies were conducted in accordance with the local legislation and institutional requirements. The participants provided their written informed consent to participate in this study. Written informed consent was obtained from the individual(s) for the publication of any potentially identifiable images or data included in this article.

## Author contributions

JL: Conceptualization, Data curation, Formal analysis, Funding acquisition, Investigation, Methodology, Project administration, Resources, Software, Supervision, Validation, Visualization, Writing – original draft, Writing – review & editing.
